# Synergistic Interactions Between Plant Growth-Promoting Bacteria and *Trichoderma virens* Enhance *Arabidopsis* Growth and Suppress *Fusarium brachygibbosum*

**DOI:** 10.3390/plants15142234

**Published:** 2026-07-22

**Authors:** Paulina Guzmán-Guzmán, María D. Ramírez-Ramos, Ma. del Carmen Orozco-Mosqueda, Juan Francisco Jiménez-Bremont, Paola Fincheira, Mauricio Schoebitz, Eduardo Valencia-Cantero, Gustavo Santoyo

**Affiliations:** 1 Department of Chemical and Biochemical Engineering, Instituto Tecnológico de Morelia, Tecnológico Nacional de México, Morelia 58000, Mexico; paulina.gg@morelia.tecnm.mx; 2 Institute of Chemical and Biological Research, Universidad Michoacana de San Nicolás de Hidalgo, Morelia 58000, Mexico; 1720375f@umich.mx (M.D.R.-R.); eduardo.cantero@umich.mx (E.V.-C.); 3 Department of Biochemical and Environmental Engineering, Instituto Tecnológico de Celaya, Tecnológico Nacional de México, Celaya 38010, Mexico; carmen.orozco@itcelaya.edu.mx; 4 Laboratorio de Biotecnología Molecular de Plantas, División de Biología Molecular, Instituto Potosino de Investigación Científica y Tecnológica, A. C., San Luis Potosí 78216, Mexico; jbremont@ipicyt.edu.mx; 5 Departamento de Ingeniería Química, Facultad de Ingeniería y Ciencias, Universidad de La Frontera, Temuco 4811230, Chile; paola.fincheira@ufrontera.cl; 6 Department of Soil Science and Natural Resources, Faculty of Agronomy, Universidad de Concepción, Concepción P.O. Box 160 C, Chile; mschoebitz@udec.cl; 7 Center of Biotechnology, Universidad de Concepción, Concepción P.O. Box 160 C, Chile; 8 Soil and Ecosystem Functions Research Center of Chile (CISFECh), Valdivia 5090000, Chile

**Keywords:** plant growth-promoting bacteria (PGPB), *Trichoderma virens*, *Fusarium brachygibbosum*, microbial consortia, sustainable agriculture

## Abstract

*Trichoderma virens* and plant growth-promoting bacteria (PGPB) are well-known agents that promote plant development and control pathogens. This study assessed the compatibility, biocontrol potential, and plant growth promotion of *T. virens* in combination with four PGPB strains (*Pseudomonas fluorescens* UM270, *Rouxiella badensis* SER3, *Bacillus velezensis* AF12, and *Bacillus halotolerans* AF23) against *Fusarium brachygibbosum* and *Arabidopsis thaliana*. The results showed that single inoculations significantly inhibited the growth of *F. brachygibbosum* by the 7th day of confrontation. However, co-inoculating *T. virens* with PGPB exhibited synergistic effects on the inhibition percentages for the consortia Tv + UM270 (48.94%), Tv + AF12 (67.04%), and Tv + SER3 (78.63%). Plant assays demonstrated that most microorganisms enhanced root development and plant height, with UM270 having the strongest beneficial effect. Expression analysis of *T. virens* effector genes (*sm1*, *tvsep3*, and *tvhydii1*) indicated early induction of *tvhydii1* in the condition of Fb + AF12 at day 3, while *sm1* was downregulated. No significant changes in the expression of these genes were detected during interaction with *A. thaliana* and PGPB. These findings demonstrate that *T. virens*–PGPB can simultaneously promote plant growth and suppress pathogens, with effector genes such as *tvhydii1* contributing to these interactions, highlighting their potential for sustainable agriculture.

## 1. Introduction

The plant microbiome plays a crucial role in promoting plant health, growth, and productivity [1]. Beneficial microorganisms, such as plant growth-promoting microorganisms associated with plants, contribute to nutrient acquisition, stress tolerance, and protection against pathogens, ultimately enhancing crop yields and sustainability in agriculture [2]. However, intensive agricultural practices often rely heavily on agrochemicals, including synthetic fertilizers and pesticides, which can negatively affect soil health, reduce microbial diversity, and disrupt beneficial plant–microbe interactions [3]. This has led to a growing interest in using beneficial microorganisms as environmentally friendly bioinoculants to improve crop performance and reduce dependency on chemical inputs, where rhizosphere interactions can be harnessed for a more sustainable agriculture, helping to transition from traditional chemical-intensive methods to nature-based solutions.

Complex relationships between plants and soil microbes can be harnessed in different ways, including soil microbial transplantation, in situ manipulation and regulation of soil microbes, exploitation of core beneficial microorganisms, and the development of synthetic microbial communities or SynComs [4].

The plant microbiome is composed of different types of microorganisms, including fungi and bacteria [5]. Among the most common soil inhabitants and plant microbiome components are *Trichoderma* species [6], which [7,8] have been known to modulate the plant-associated microorganisms to the plant’s benefit [7,8,9], improving health and protection against pathogens. Among the *Trichoderma* species, *Trichoderma virens* is known to be an excellent biocontrol agent [10] against several plant pathogens, and it is also a well-known plant growth-promoter [11]. For example, *T. virens* XZ11-1 inhibited the growth of several fungal plant pathogens including *F. oxysporum* f.sp. *cubense* 4 (FOC4), *Fusarium oxysporum* f.sp. *lycopersici*, *Colletotrichum fragariae*, *C. gloeosporoides* and *Botrytis cinerea*, and improved overall banana plant health and growth [12]. *T. virens* ZT05 is a mycoparasite that inhibits the growth of *R. solani* due to its ability to produce antagonistic secondary metabolites and induce oxidative stress in its fungal prey [13]. *T. virens* Gv29-8 has shown mycoparasitic activity against the plant pathogen *R. solani* AG5 [14].

Additionally, plant growth-promoting bacteria (PGPB) are used as biocontrol agents and plant biostimulants. For example, *Pseudomonas aeruginosa* Ld-8 had antagonistic capacity against different plant pathogens such as *F. oxysporum*, *B. cinerea*, *Botryosphaeria dothidea,* and *F. fujikuroi*, and improved the growth of *Lilium davidii* plants [15]. The PGPB *P. fluorescens* UM270 has shown biocontrol properties against different plant pathogens such as *B. cinerea*, *R. solani*, and *Fusarium* spp. [16], and *Rouxiella badensis* SER3 inhibited the growth of different post-harvest pathogens, including *F. brachygibbosum* [17]. The PGPBs *B. halotolerans* AF23 and *B. velezensis* AF12 were confronted with *F. brachygibbosum* [17]. However, their inhibitory effect on the phytopathogen was better when in consortium with *Trichoderma atroviride*.

Most studies on beneficial plant-associated microorganisms have focused on the interactions between a single microorganism and the plant and/or the pathogen. However, several studies have shown that the combination of two or more beneficial microorganisms can generate synergistic effects that enhance their performance as biocontrol agents or biostimulants. These combinations may include different species of *Trichoderma* and PGPB, whose combined application more closely resembles the field conditions. For example, the co-inoculation of *T. longibrachiatum* with *B. aryabhattai* that synergistically improved rice growth and resistance to abiotic stress [18], or the combination of *Trichoderma* sp. and *Bacillus* sp. B16 reduced disease severity caused by *F. oxysporum* f.sp. *capae* in shallot plants [19].

Most of the work related to consortia between microorganisms focuses on their ability to inhibit plant pathogens’ growth or plant stimulation, leaving aside the molecular mechanism by which this interaction could occur. Molecular dialogue occurs between beneficial or pathogenic microorganisms and plants during their interactions [20,21], and effector proteins from *T. virens* such as Sm1 [22,23] and Tvhydii1 from the strain Gv29-8 [14] could be mediating the fungus’s interactions with other organisms.

Thus, because *T. virens* Gv29-8 has shown mycoparasitic activity, and effector-coding genes from this strain are induced in the presence of a plant pathogen and the plant *A. thaliana* [14], it was decided to evaluate its interactions with four plant growth-promoting bacteria, with the aim of identifying at least one synergistic consortium with the ability to promote plant growth and/or the ability to suppress fungal pathogens, and to elucidate if the beneficial microorganisms interaction could be mediated by *T. virens* Gv29-8 effector proteins.

## 2. Results

### 2.1. Evaluation of T. virens–PGPB Compatibility

Before assembling *T. virens*–PGPB combinations, it was first assessed whether both the fungus and the bacteria could grow together in the medium used for biocontrol and growth-promotion assays, and whether *T. virens* germination from conidia or growth from mycelia would be affected by the presence of the tested PGPBs. *T. virens* was not inhibited by any of the four PGPBs evaluated; however, the presence of *B. velezensis* AF12 and *B. halotolerans* AF23 slightly restricted fungal growth (Figure S1).

**Figure 1:**
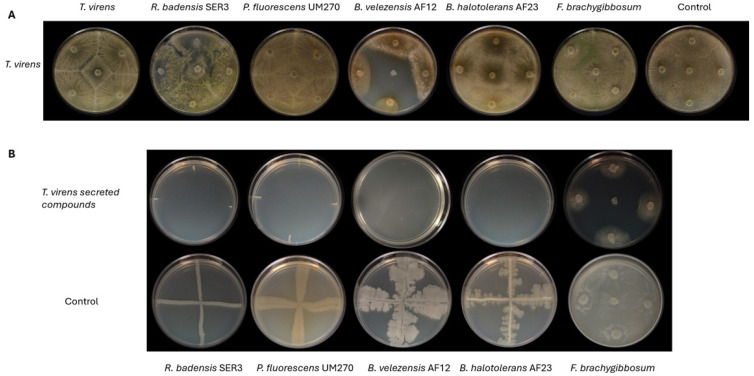
**Inhibitory effects of secreted microbial compounds**. (**A**) Growth of *T. virens* in the presence of secreted compounds from each microorganism (PGPBs and *F. brachigibbosum*), with *T. virens* growing without secreted compounds used as a control. (**B**) Microbial growth in the presence of secreted compounds from *T. virens* (**upper line**), with the corresponding controls grown without secreted compounds (**bottom line**). The experiment was performed three times with similar results. Photographs are representative of the end of the experiment.

Given the observed slight growth inhibition of *T. virens* by some of the PGPBs selected for this study, it was then determined whether secreted compounds from either microorganism or from the plant pathogen *F. brachygibbosum* could inhibit the growth of the fungus or the bacteria, thereby potentially hindering consortium formation [17,24]. As shown in Figure 1A, when *T. virens* grows over the bacteria- and pathogen-secreted compounds, only AF12’s secreted compounds inhibited fungal growth to some extent. Nonetheless, the compounds secreted by *T. virens* inhibited the growth of all PGPBs and pathogens (Figure 1B).

Both experiments demonstrated that *T. virens* can grow and germinate in the presence of each tested PGPB, although some degree of mutual inhibition was observed in certain combinations. Since the biocontrol and plant growth-promotion assays require simultaneous inoculation of the microorganisms (Figure S1), we further observed that, during co-inoculation, both types of microorganisms were able to grow in the presence of each other. Overall, these results indicate that consortium formation is feasible and that there would be no major constraints for performing the planned experiments.

### 2.2. Antagonism Bioassays of T. virens–PGPB Against F. brachygibbosum

*T. virens* is a well-known mycoparasite and biocontrol agent of different plant pathogens, including *Fusarium* species [25]. Plant growth-promoting bacteria are also known to inhibit the growth of several phytopathogens and are used as biocontrol agents as well [26].

**Figure 2:**
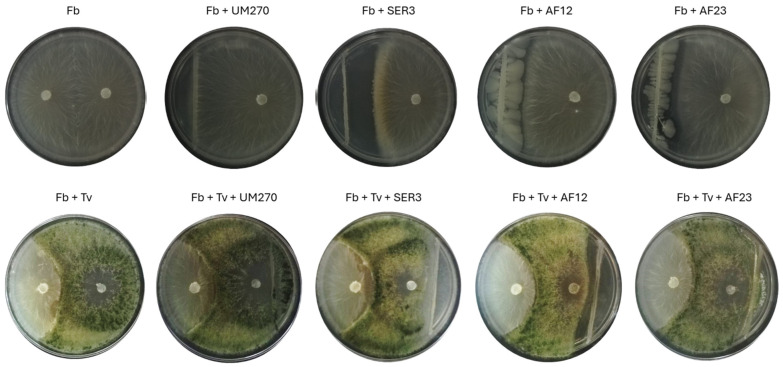
**Biocontrol activity of *Trichoderma virens* and PGPB against the phytopathogen *Fusarium brachygibbosum*.** *T. virens* and PGPB biocontrol over the phytopathogen *F. brachygibbosum* at the 7th day of confrontation. The experiment was performed three independent times; images are representative of the experiments. Fb, *F. brachygibbosum*; Tv, *T. virens*; UM270, *P. fluorescens* UM270; SER3, *R. badensis* SER3; AF12, *B. velezensis* AF12; AF23, *B. halotolerans* AF23.

The ability of PGPBs and *T. virens* against the post-harvest pathogen *F. brachygibbosum* [27]. Biocontrol assays were carried out in PDA medium as described in Section 4. Figure 2 shows representative photographs of the experiment on the 7th day of confrontation.

**Figure 3:**
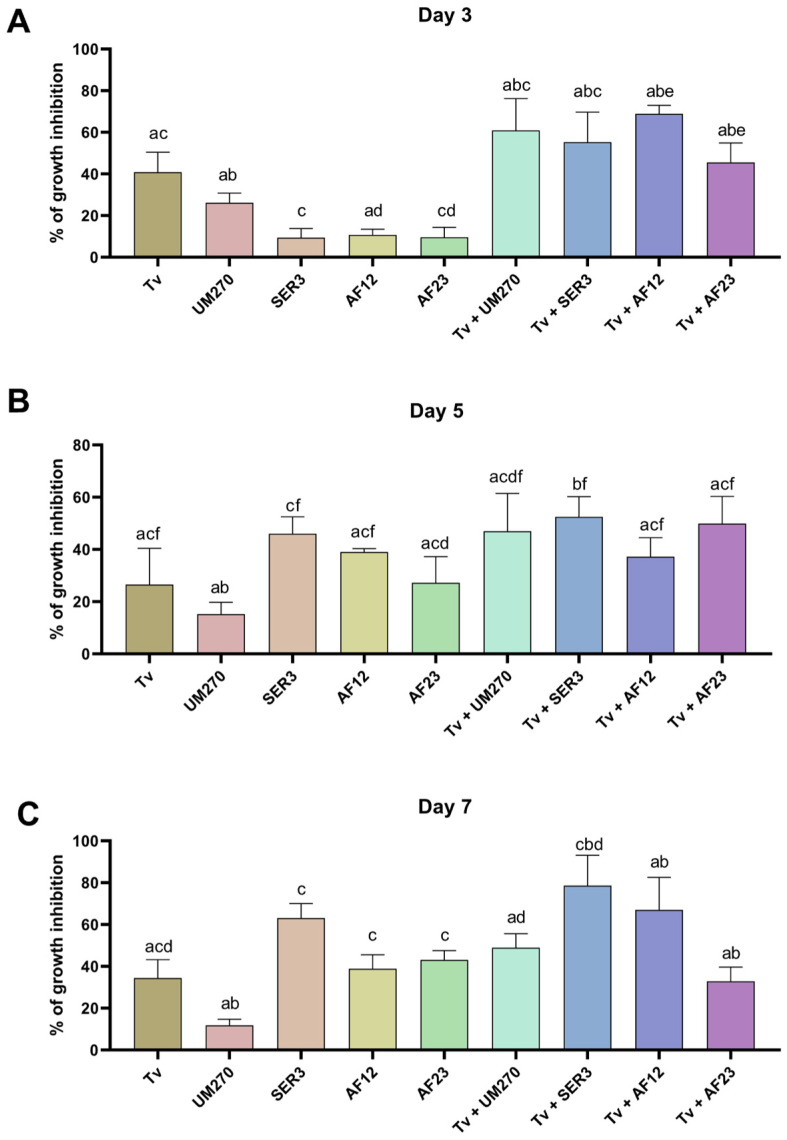
***F. brachygibbosum* growth inhibition by *T. virens*–PGPB consortia.** Percentage of growth inhibition of the phytopathogen *F. brachygibbosum* by microorganisms alone or in combination, at 3 (**A**), 5 (**B**), and 7 (**C**) days of interaction. The experiment was performed three independent times. One-way ANOVA with Tukey post-hoc test for comparison of treatments; different letters above bars indicate statistical significance at *p* < 0.05; bars indicate SEM. Fb, *F. brachygibbosum*; Tv, *T. virens*; UM270, *P. fluorescens* UM270; SER3, *R. badensis* SER3; AF12, *B. velezensis* AF12; AF23, *B. halotolerans* AF23.

On day 3 of confrontation (Figure 3A), *T. virens* (Tv) showed the highest level of inhibition of the pathogen growth (40.85%), followed by *P. fluorescens* (Ps UM270) with 26.22% inhibition. Whereas *R. badensis* (Rb SER3), *B. velezensis* (Bv AF12), and *B. halotolerans* (Bh AF23) showed lower percentages of growth inhibition (9.37%, 10.67%, and 9.59%, respectively; *p* < 0.05). On day 5 (Figure 3B), Tv inhibited pathogen growth by 26.60%, whereas Rb SER3 and Bv AF12 displayed higher inhibition levels (46.03% and 39.04%, respectively), and Bh AF23 showed 27.27% inhibition (*p* < 0.05).

Notably, the consortia exhibited greater inhibition of pathogen growth on day 3, with Tv + Bv AF12 and Tv + Ps UM270 showing the highest inhibition levels (68.85% and 60.94%, respectively), followed by Tv + Rb SER3 and Tv + Bh AF23 (55.31% and 45.52%, respectively). However, at 5 days of confrontation, consortia inhibited the pathogen’s growth up to 52.48% by Tv + Rb SER3. Tv + Ps UM270 inhibited *F. brachygibbosum* to 46.99%, Tv + Bv AF12 to 37.22% and Tv + Bh AF23 to 49.88% (*p* < 0.05; Figure 3B).

At the end of the experiment, on day 7 of confrontation (Figure 2 and Figure 3C), the individual treatments showed diverse levels of inhibition of the pathogenic fungus. Rb SER3 exhibited the highest inhibition (63.11%), followed by Bh AF23 (43.16%), Bv AF12 (38.84%), Tv (34.48%), and Ps UM270 (11.78%) (Figure 3C). Most Tv-PGPB combinations showed higher inhibition percentages than the individual microorganisms at the end of the experiment. Tv + Ps UM270, Tv + Bv AF12, and Tv + Rb SER3 inhibited pathogen growth by 48.94%, 67.04%, and 78.63%, respectively. In contrast, the Tv + Bh AF23 combination showed 32.91% inhibition, which was lower than that observed for bacteria Bh AF23 alone, indicating that this consortium did not improve pathogen growth inhibition (Figure 3C).

Taken all together, our results show that, while the beneficial microorganisms alone are capable of inhibiting *F. brachygibbosum*’s growth, such as Tv, SER3, AF12, and AF23 by the end of the experiment, the Tv + PGPB combination resulted in a greater pathogen-growth inhibition since the 3rd day of confrontation, especially with Tv + AF12; nonetheless, the combination Tv + SER3 was the best one on the 7th day of confrontation, with 78.63% of growth inhibition of the pathogen. These results also show that, in pairing some of the PGPBs with *T. virens*, the inhibition is better than the microorganisms alone; this is the case with Tv + UM270, Tv + AF12, and Tv + SER3, suggesting that their interactions may have a potentiated effect, and their effect increases over time (Figure 3). Nonetheless, in the case of Tv + AF23, the percentage of inhibition of the consortium was not statistically different (*p* > 0.05) from that of the microorganisms alone on the 5th and 7th days of confrontation, and was less than that of the other consortia, suggesting that this combination of microorganisms may not exhibit a synergistic interaction (Figure 3). Overall, the results show that, although inhibition of *F. brachygibbosum* growth was observed with individual beneficial microorganisms, a greater degree of pathogen inhibition was achieved with most Tv + PGPB combinations.

### 2.3. Plant Growth Promotion by T. virens–PGPB Interactions

The fungus *T. virens* [28] and the bacterium *P. fluorescens* UM270 [16] are microorganisms known to have a positive effect on plant growth, increasing plant biomass and yield. *In vitro* plant growth promotion assays were performed using beneficial microorganisms alone or in combination with *A. thaliana* plants, and non-inoculated plants were used as controls. Primary root length, number of lateral roots, plant height, and fresh and dry weights were measured 3 and 5 days after interaction.

**Figure 4:**
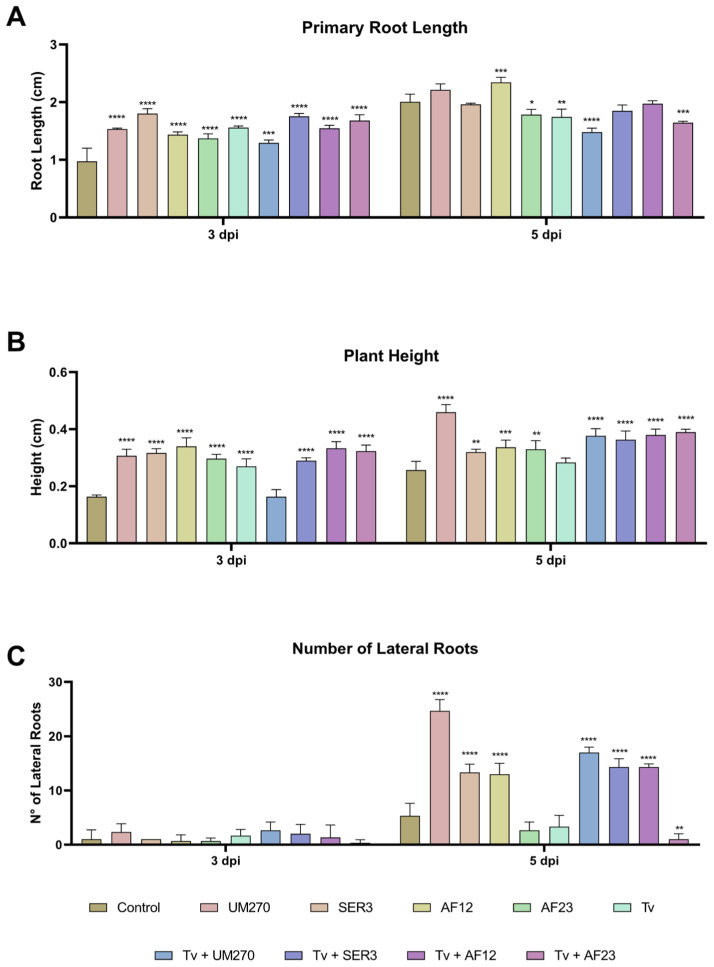
**Plant growth-promotion of *A. thaliana* by *T. virens*–PGPB consortia.** Effect of the inoculation of the microorganisms alone or in combination on the plant primary root (**A**), plant height (**B**), and number of lateral roots formed (**C**) at 3 and 5 days of interaction (dpi). Two-way ANOVA with Dunnet test of mean comparison; * *p* < 0.05; ** *p* < 0.01; *** *p* < 0.001; **** *p* < 0.0001 compared to control. Tv, *T. virens*; UM270, *P. fluorescens* UM270; SER3, *R. badensis* SER3; AF12, *B. velezensis* AF12; AF23, *B. halotolerans* AF23.

Figure 4A shows the primary root length of *A. thaliana* under each treatment. On day 3 of interaction, primary root length was greater in all treatments, including both individual microorganisms and their combinations, compared with the uninoculated control. However, on day 5, only AF12 promoted an increase in root length (16.78%) compared with the control plants (Figure 4A). In contrast, plants interacting with AF23, Tv, Tv + UM270, and Tv + AF23 exhibited significantly shorter primary roots than the control plants.

On the 3rd day of interaction, plants in the presence of beneficial microorganisms alone or in consortia, except for Tv + UM270, were taller than control plants (*p* < 0.0001; Figure 4B). On the 5th day, plants in interaction with either the microorganism consortia or microorganisms alone, except for Tv, were taller than plants growing alone (Figure 4B). It is noteworthy that plants in the presence of UM270 showed a 79.22% increase in height on the 5th day (*p* < 0.0001; Figure 4B), being the treatment that had the most positive effect on plant height. None of the treatments had a detrimental effect on plant height (Figure 4B).

Regarding the number of lateral roots (Figure 4C), on the 3rd day of interaction, there was no statistical difference between plants growing in the presence of the beneficial microorganisms or the consortia (*p* > 0.05), compared to plants growing alone. However, on the 5th day of interaction, plants co-inoculated with UM270, SER3, AF12, Tv + UM270, Tv + SER3, and Tv + AF12 showed more lateral roots than plants growing alone (Figure 4C), noting that plants in interaction with UM270 alone showed 362.5% more lateral roots than control plants (*p* < 0.0001; Figure 4C). Plants growing with AF23 or Tv showed no statistical differences compared to control plants (*p* > 0.05), and plants growing with Tv + AF23 showed 81.25% fewer lateral roots than plants growing alone (*p* < 0.01; Figure 4C).

According to these results, plant growth-promotion was induced particularly with UM270 alone, although the other treatments also showed growth-promotion, except for the consortium Tv + AF23, which decreased the number of lateral roots and the length of the primary root, suggesting that this combination of microorganisms may be detrimental to the plant root system.

**Figure 5:**
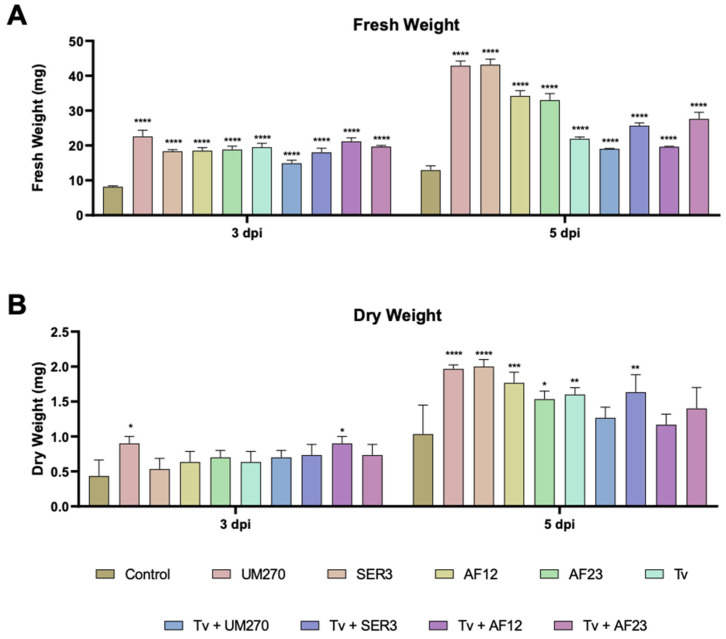
**Plant biomass.** Effect of the inoculation of the microorganisms alone or in combination on the plant fresh (**A**) and dry weight (**B**) at 3 and 5 days of interaction (dpi). Two-way ANOVA with Dunnett test of mean comparison; * *p* < 0.05; ** *p* < 0.01; *** *p* < 0.001; **** *p* < 0.0001 compared to the control. Tv, *T. virens*; UM270, *P. fluorescens* UM270; SER3, *R. badensis* SER3; AF12, *B. velezensis* AF12; AF23, *B. halotolerans* AF23.

Figure 5A shows *A. thaliana* fresh weight. On the 3rd and 5th day of interaction, plants growing in the presence of either the microorganisms alone or their consortia had an increase in fresh weight compared to plants growing alone (*p* < 0.0001). Notably, on the 5th day of interaction, plants growing with UM270, SER3, AF12, and AF23 showed an increase of 231.7%, 233.76%, 164.69%, and 155.41%, respectively, compared to the control plants (*p* < 0.0001; Figure 5A).

Regarding the dry weight of *A. thaliana*, Figure 5B shows that, on the 3rd day of interaction, only plants growing in the presence of UM270 and Tv + AF12 showed an increase in dry weight in comparison to control plants (*p* < 0.05), and no statistically significant difference was found between the other treatments and control plants (*p* > 0.05). Nonetheless, on the 5th day of interaction, plants growing with UM270, SER3, AF12, AF23, Tv, and Tv + SER3 showed a statistically significant increase in dry weight compared to plants growing alone (Figure 5B). Plants growing with UM270 and SER3 showed 90.32% and 93.55% increases in dry weight, compared to plants growing alone (*p* < 0.0001), and no detrimental effects were found among the treatments.

Taken together, our results indicate that the highest increases in plant biomass were achieved with individual bacterial treatments, especially UM270 and SER3, followed by *T. virens*-containing consortia, compared to uninoculated plants.

### 2.4. Expression Analysis of T. virens Effector Coding Genes sm1, tvsep3, and tvhydii1

Microorganisms like *Trichoderma* and PGPBs use different types of molecules, like effector proteins, to communicate with nearby organisms, mediating interactions to establish beneficial or detrimental relationships with them [29].

**Figure 6:**
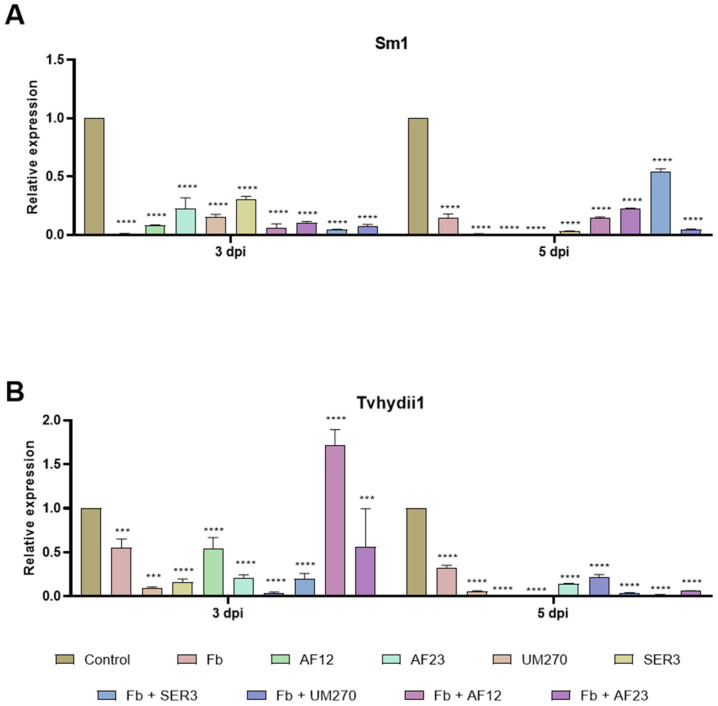
**Relative gene expression of *sm1* and *tvhydii1* in the biocontrol assays.** Gene expression of *sm1* (**A**) and *tvhydii1* (**B**) from *T. virens* during confrontation with the pathogen *F. brachygibbosum* and in co-inoculation with each PGPB. Two-way ANOVA with the Dunnett test of mean comparison; *** *p* < 0.001; **** *p* < 0.0001 compared to the control. Fb, *F. brachygibbosum*; UM270, *P. fluorescens* UM270; SER3, *R. badensis* SER3; AF12, *B. velezensis* AF12; AF23, *B. halotolerans* AF23.

**Figure 7:**
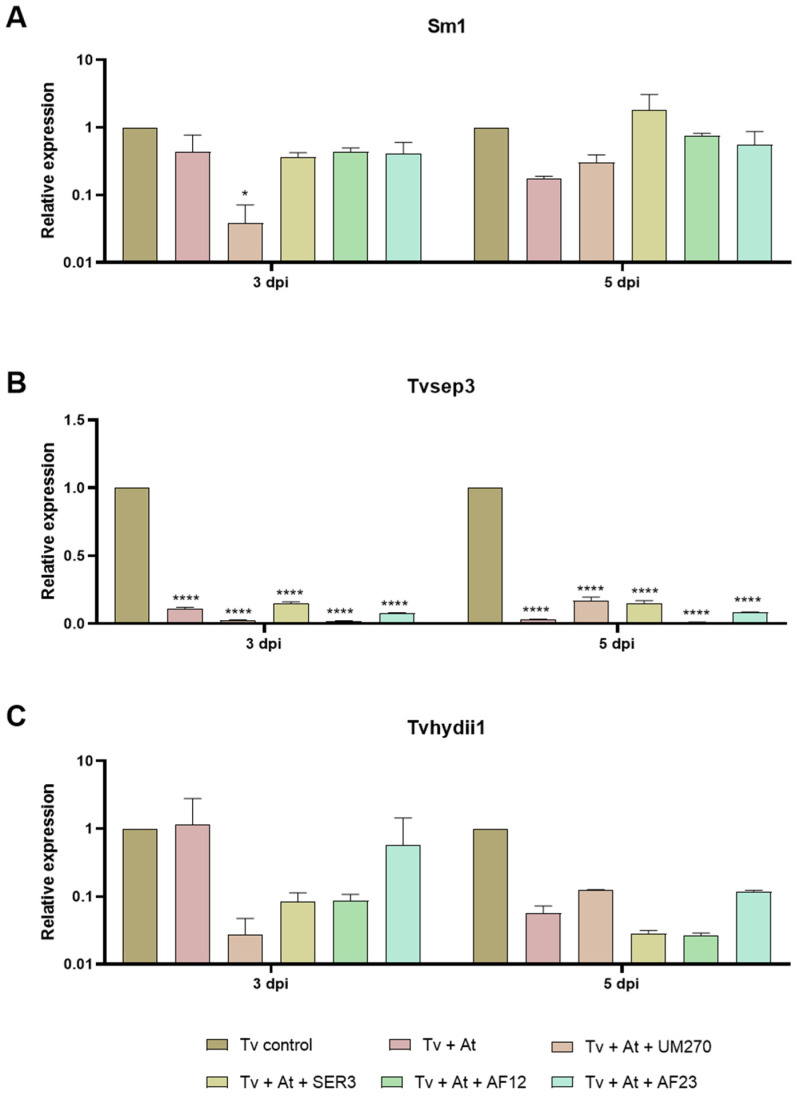
**Relative gene expression of *sm1, tvsep3* and *tvhydii1* in the plant growth-promotion assays.** Gene expression of *sm1* (**A**), *tvsep3* (**B**), and *tvhydii1* (**C**) from *T. virens* during the beneficial interaction with *A. thaliana* and in co-inoculation with each PGPB. Two-way ANOVA with the Dunnett test of mean comparison; * *p* < 0.05; **** *p* < 0.0001 compared to the control. Tv, *T. virens*; At, *A. thaliana;* UM270, *P. fluorescens* UM270; SER3, *R. badensis* SER3; AF12, *B. velezensis* AF12; AF23, *B. halotolerans* AF23.

To determine whether some of the effectors from *T. virens* previously identified [14] could be involved in its interactions with host organisms, mycelia of *T. virens* were collected during confrontation with *F. brachygibbosum*, during its beneficial interaction with *A. thaliana*, and in combination with each PGPB tested in this study. Expression of the effector-coding genes sm1, tvhydii1, and tvsep3 was then assessed on days 3 and 5 of the interactions. Figure 6 shows relative gene expression of *sm1* and *tvhydii1* during the biocontrol assays, and Figure 7 shows the relative expression of the effector-coding genes during the beneficial interaction with *A. thaliana*.

As shown in Figure 6A, *sm1* relative expression was downregulated in the presence of the pathogen and the PGPBs compared to its expression growing alone (*p* < 0.0001) at both the 3rd and 5th days of confrontation, and *tvhydii1* relative expression was induced only on the 3rd day of interaction in the presence of the pathogen and AF12 (Fb + AF12; 1.7 ± 0.1 fold change, *p* < 0.0001; Figure 6B), while, in the other treatments, its expression was downregulated compared to the control conditions. By the 5th day of confrontation, *tvhydii1* expression was downregulated in the presence of the pathogen and the PGPBs tested, compared to the control conditions (*p* < 0.0001) (Figure 6B). These results suggest that *sm1* from *T. virens* may not be involved in its interaction with the PGPBs used or its confrontation with *F. brachygibbosum*. The gene *tvhydii1* may be involved only at early stages of the interaction with AF12 and the pathogen. In the conditions of the biocontrol experiment, no detectable gene expression of *tvsep3* was observed. Further confirmation is needed to discard improper technique or sample manipulations or to determine if this gene may not be active in the presence of the PGPBs or the pathogen tested in this study.

During the beneficial interaction of *T. virens* with *A. thaliana*, the relative expression of gene *sm1* was not statistically different from the control (*p* > 0.05) in the presence of the plant or any PGPB used (Figure 7A), rather its expression was downregulated in the consortium with UM270 on the 3rd day of interaction (0.038 ± 0.019-fold change, *p* < 0.05, compared to control; Figure 7A). Figure 7B shows that the relative expression of *tvsep3* was downregulated at both the 3rd and 5th days of interaction in the presence of the plant and the PGPBs used in this study (*p* < 0.0001). No statistically significant differences were detected in the relative expression of *tvhydii1* compared to the control conditions (*p* < 0.05) across all treatments (Figure 7C). These results suggest that the effectors selected from *T. virens* may not be required during their beneficial interaction with *A. thaliana* or either of the PGPBs tested.

## 3. Discussion

In natural conditions, plant-associated microorganisms interact in complex networks that may lead to synergistic or non-synergistic effects on plants and on their biocontrol traits [30,31,32]. First, in this study, we verified the compatibility of the tested strains for growth in co-culture. Although *T. virens*-secreted compounds inhibited the PGPBs used in this study (Figure 1), there was no growth inhibition, only some growth constriction when both microorganisms were inoculated at the same time (Figure S1). This finding was relevant to the present study, as strain compatibility is important for the formation of microbial consortia, and the nature of their interaction may strongly influence their effects on plant interactions and their ability to act as biocontrol agents [30,33,34]. However, experiments such as growth inhibition by secondary metabolites and/or co-inoculation in field conditions are necessary to determine the possible implications of microorganisms’ consortia application in the field, since soil conditions may render different results due to the complex nature of soil and different ecosystems [31,32,35], and results may not be as desired or as observed in controlled conditions. Additionally, the production of secondary metabolites is influenced by environmental conditions [36], and the fungal growth medium is a relevant factor in plant growth induction [37].

Our results of microbial compatibility, specifically the antagonism of the consortium formed between Tv and AF23, show the importance of effective harnessing of microbiome components to ensure desired beneficial traits over plants, leading research in the field of microbiome engineering to consider all aspects of how microorganisms interact with each other beforehand, and how they can help each other to adapt to environmental conditions, or in other cases, how they interfere between them and fail to establish a positive interaction [38].

Biocontrol assays were performed with *T. virens* (Tv) and PGPBs applied individually and in combination against the postharvest pathogen *F. brachygibbosum* (Figure 2 and Figure 3). Each beneficial microorganism alone inhibited pathogen growth to some extent at both 5 and 7 days of confrontation, with UM270 being the least effective overall (Figure 3C) and SER3 the most effective at the end of the experiment. However, the consortia formed by Tv + PGPB exhibited inhibitory activity from the early stages of the experiment (Figure 3A), particularly the Tv + AF12 combination. It is also worth noting that the Tv + SER3, Tv + UM270, and Tv + AF12 consortia were more effective at inhibiting *F. brachygibbosum* growth than the individual organisms. Moreover, although the inhibitory effect increased over time for the Tv + SER3, Tv + UM270, and Tv + AF12 consortia, Tv + SER3 consistently showed the strongest activity at the end of the experiment. Synergistic antagonistic activity between the PGPB and Tv was specifically observed against *Fusarium*. The combinations Tv + SER3 and Tv + AF12 showed increases of 10.13% and 33.64%, respectively, at 3 dpi, whereas Tv + UM270 exhibited synergistic effects at 5 and 7 dpi, with increases of 12.28% and 5.8%, respectively. These results suggest that different mechanisms of action may be involved in pathogen suppression, and that the way *T. virens* interacts with each PGPB differs, leading to variable biocontrol outcomes.

These results are consistent with studies that indicate that the combination of *Trichoderma* species and PGPB performs better than either organism alone as biocontrol agents. The supernatant obtained from the co-culture of *T. virens* Gl006 and *B. velezensis* Bs006 reduced the disease severity in gooseberry plants caused by *F. oxysporum* f. sp. *physalis* to 72%, and its effect was better than the effect of the supernatant of the microorganisms alone [39]. Izquierdo-García et al. [39] determined that the consortium composed of these microorganisms had a synergistic effect, making them good candidates as biocontrol agents, and that these species were also compatible. In our study, the best combination of microorganisms was Tv + SER3, which inhibited the pathogen’s growth on the 7th day of confrontation, followed by Tv + AF12 and Tv + UM270. These results are in accordance with our previous work, where *T. atroviride* and SER3 were also excellent at inhibiting Fb [17].

It was also found that the effect of the Tv + AF23 consortium on pathogen growth was not synergistic, as shown in Figure 3, since no significant differences were observed compared with Tv or AF23 alone. It is suggested that, in this case, mutual inhibition may play a more important role in their interaction than in the other consortia formed, as both microorganisms are strong producers of secondary metabolites that can inhibit each other [40], and evidence shows that *T. virens* G-41 strongly inhibits several *Bacillus* and *Pseudomonas* isolates [41]. Since our consortia included two different *Bacillus* species, and only the consortium formed with *B. halotolerans* showed no improved effect compared to the microorganisms alone and a strong mutual inhibition by secondary metabolites (Figure 1B), we speculate that this specific *Bacillus* strain, *B. halotolerans* AF23, may be more sensitive to some of *T. virens*’s metabolites and vice versa, than *B. velezensis* AF12 or any of the other PGPBs tested, in such a way that the communication between both microorganisms fails to establish a positive interaction, and neither microorganism can exert their biocontrol traits effectively against *F. brachygibbosum*.

Accordingly, the combination of *T. virens* and *B. halotolerans* AF23 may not be a suitable biocontrol consortium under the conditions tested. However, different outcomes may be obtained against other plant pathogens. Further investigation is required to test this hypothesis, as these interactions are highly strain- and pathogen-specific.

Regarding plant growth promotion, most treatments had a positive effect on the primary root length of *A. thaliana*. On day 3 of interaction, all microorganisms, applied either individually or in combination, increased primary root length; however, SER3 and Tv + SER3 were the most effective treatments (Figure 4A). In contrast, on day 5 of interaction, only AF12 had a positive effect on primary root length, and a reduction in primary root length was observed in the AF23, Tv, Tv + SER3, and Tv + AF23 treatments. Plant root development is regulated by a complex crosstalk signaling between different phytohormones, including auxin, cytokinins, gibberellins, abscisic acid, brassinosteroids, and salicylic acid, and changes in hormone concentration can lead to developmental defects [36,42]. Plant-associated microorganisms can produce phytohormone-like compounds, such as auxins, ethylene, and salicylic acid, affecting the plant root system, increasing lateral root formation, primary root length, and root hairs, including microorganisms like *Trichoderma* spp. [43] and different PGPB [44,45].

However, some PGPB, such as *Bacillus* sp. LZR216 and *Azospirillum brasilense* Sp245, inhibit primary root length and at the same time enhance lateral root formation in *A. thaliana* and wheat plants, respectively (revised in [44]), via auxin production and increasing gene expression of auxin biosynthesis genes. It is suggested that plant-associated microorganisms with the ability to produce auxins or modulate auxin biosynthesis in plants can lead to the root architectural changes observed in this experiment. The primary root of *A. thaliana* was shorter on day 5 of interaction with the tested microorganisms, while the number of lateral roots increased, suggesting that the consortia may affect auxin signaling in the plant.

The number of lateral roots increased on day 5 of interaction in most treatments (Figure 4C), with the most pronounced effect observed for UM270 alone, followed by the Tv + UM270 consortium. SER3, AF12, Tv + SER3, and Tv + AF12 also promoted lateral root formation; however, Tv + AF23 was the only treatment that had a negative effect, as plants exhibited fewer lateral roots than the control. In addition, plants grown in the presence of most microorganisms were taller than control plants (Figure 4B), with UM270 alone being the most effective biostimulant at day 5 of interaction. Regarding plant growth promotion, synergistic effects were most pronounced for root length in the combinations of Tv with SER3, AF12, and AF23, with increases ranging from 15.63% to 58.82% (see Supplementary Data for detailed results).

There is evidence suggesting that some *Trichoderma* species, like *T. koningii*, could have detrimental effects on plants and on other indirect targets, such as plant-feeding insects (see [46] for a complete review on this subject). However, it is not completely understood how this can occur, but the main idea is that it may have to do with the modulation of the SA pathway in plants [47,48]. Further investigation on this subject is needed to elucidate why, in our experimental conditions, the combination Tv + AF23 may have detrimental effects on plants; however, it is known that root architecture is regulated by several molecules, such as small peptides, and microbial interactions like mycorrhiza and rhizobia, can alter root architecture as well [49], so it is possible that the antagonistic interaction between Tv and AF23 could be altering root development by specific secondary metabolites from these microorganisms, interfering with plant hormonal signaling [49].

Taken together, these results indicate that UM270, alone or in combination with *Tv*, acts as an effective biostimulant of the plant’s secondary root system and overall growth, whereas AF12 primarily enhances primary root development. This is a desirable trait, as a more extensive secondary root system together with a longer primary root are key determinants of efficient water and nutrient uptake [50,51] and both represent important plant growth-promoting traits of microorganisms used as biostimulants.

The microorganisms alone or their combinations had no negative effect on plant biomass (Figure 5). Nonetheless, in this case, the microorganisms alone had a better effect than their combination with Tv, with UM270 and SER3 standing out. *P. fluorescens* UM270 has been characterized as an excellent plant growth-promoter of maize, bean, tomato, among others [16] and of *A. thaliana* [17]. Thus, our results are in accordance with what has been reported of this plant-beneficial microorganism.

Although several studies have shown that consortia of plant-beneficial microorganisms generally outperform individual strains, it was not the case in the present study for some of the evaluated combinations and parameters in the biocontrol and plant growth-promotion assays. Interestingly, the combination of *T. virens* with the PGPB *B. halotolerans* AF23 had no statistically significant differences regarding inhibition of *F. brachygibbosum* compared to the effect of the microorganisms alone, and, in the case of the plant assays, Tv + AF23 had a negative effect on primary root length and the number of lateral roots of *A. thaliana*. These observations suggest that this combination may not work properly if used as plant biostimulants or as biocontrol agents, and it may be due to the mutual inhibition effect mediated by secondary metabolites [40,41]. On the other hand, the combinations Tv + UM270, Tv + SER3, and TV + AF12 outperformed the microorganisms alone at inhibiting *F. brachygibbosum*’s growth in our biocontrol assays, suggesting a synergistic interaction of these plant-beneficial microbes as biocontrol agents.

Communication between microorganisms is mediated by different kinds of molecules, including effector proteins. Effector proteins can modify host physiology, as in the case of plant–pathogen interactions, favoring the establishment of the pathogen [52,53]. Nonetheless, plant beneficial microbes also use effector molecules to communicate and mediate their beneficial interactions with plants, like effectors NopJ, NopM, and NopT from *Rhizobium* sp. NGR234, which have an important role in nodulation of the legume *Crotalaria juncea*, modulating symbiosis [54]. The results of the biocontrol and plant growth promotion assays showed that the tested consortia exhibited different degrees of interaction. Therefore, we examined several effector-coding genes that may be involved in the communication between *T. virens*, PGPBs, and their hosts. The genes *sm1*, *tvsep3*, and *tvhydii1* were selected for further analysis.

Sm1, a cerato-platanin protein from *T. virens,* is a well-characterized effector, implicated in modulating the plant immune response to favor the beneficial *T. virens*–plant interaction [22,55,56], and in mycoparasitic or antagonistic interactions against fungal phytopathogens [23]. To determine if the *sm1* gene from *T. virens* could be involved in the establishment of the interactions of this fungus with *F. brachygibbosum*, with *A. thaliana,* and in consortium with each PGPB, the gene expression of *sm1* was determined during the biocontrol and the plant growth-promotion assays.

Our results regarding the relative gene expression of *sm1* during the biocontrol assays showed that this gene was downregulated in all conditions tested: in the presence of Fb and in the presence of each PGPB (Figure 6A). These results were contrary to our expectations, and to our knowledge, there is limited information available regarding its role in the mycoparasitic or biocontrol interactions of *T. virens*, as well as in its interactions with other beneficial microorganisms. Djonović et al. [22] reported that *sm1* was repressed at early time points when *T. virens* Gv29-8 was grown in medium containing *Rhizoctonia* cell walls and also observed catabolic repression of this gene in a glucose-rich medium during early stages of growth. This regulatory pattern could explain the downregulated expression observed in the present study, and a different expression profile may occur at later stages of confrontation. It is further suggested that the inhibition of *sm1* observed here may be associated with glucose-mediated repression, as previously described by [22], since PDA is a glucose-rich medium; however, this requires further confirmation.

*Sm1* or its orthologue *Epl-1* from different *Trichoderma* species have been characterized for their roles in mycoparasitism or biocontrol. For example, Liu et al. [23] reported that the fusion of Sm1 from *T. afroharzianum* with the chitinase protein Chit42 presented a synergistic effect against *B. cinerea* and *R. solani*. According to Gomes et al. [57], *Epl-1* from *T. harzianum* participates in hyphal coiling and hyphal recognition of the phytopathogen *Sclerotinia sclerotiorum*, but it is not essential for the fungus’s antagonistic ability because its deletion did not affect *T. harzianum*’s capacity to antagonize *S. sclerotiorum*, *R. solani, or F. solani*. Thus, *sm1* from *T. virens* Gv29-8 may not be essential for its interaction with *F. brachygibbosum* or with the PGPBs tested in this study.

During the beneficial interaction of *T. virens* with *A. thaliana* and the PGPBs, no significant differences were observed in the relative expression of *sm1* at any of the evaluated time points, except for the Tv + At + UM270 treatment, in which *sm1* expression was downregulated (Figure 7A). It was not expected to observe this pattern, since *sm1* from *T. virens* Gv29-8 is induced in the presence of cotton seeds in a hydroponic system [22], as well as during early stages of maize root colonization [58]. In other *Trichoderma* species, *sm1* has been reported to be expressed in the presence of macerated roots [59]. However, in a study of the transcriptomic response of two knockout strains from *T. virens* Gv29-8, *Δsm1* and *Δsir1*, during maize root colonization, contrary to what the authors expected, Sir1 had a larger role in the plant-fungus interaction than Sm1 [60].

In our work group, it was previously identified that eight potential effector-coding genes from *T. virens* [14], and two of them, *tvhydii1* and *tvsep3*, a type II hydrophobin coding gene and a serine protease coding gene, respectively, presented interesting relative expression patterns in the presence of the pathogen *R. solani* and in interaction with *A. thaliana*. *Tvhydii1* played an important role in hyphal hydrophobicity and tomato root colonization by *T. virens*.

Thus, for this work, it was of interest to test if these two genes could be involved in the interaction of *T. virens* with a different plant pathogen, and in the presence of the PGPBs used. In the case of *tvsep3*, no gene transcripts were detected in the samples collected from the biocontrol assay. The gene *tvsp1* from *T. virens* Gv29-8 encoding a serine protease was strongly induced in the presence of cell walls from *R. solani* and *P. ultimum*, but no transcript was detected when grown in medium with sucrose or glucose as carbon sources [61]. In our case, the lack of a transcript may be due to experimental conditions, and it may be inhibited by growth in a rich medium such as PDA, but further confirmation is needed. On the other hand, during the interaction with the plant and PGPBs, the relative expression of *tvsep3* was downregulated under all conditions (Figure 7B), which was unexpected. Nevertheless, Pozo et al. [61] reported that *tvsp1* deletion did not affect the fungus’s ability to colonize cotton roots; thus, *tvsep3* may not be involved in *T. virens* interaction with plants and PGPBs at the same time, or it could be downregulated due to the presence of glucose provided by the plant.

Fungal hydrophobins have several roles, mainly related to surface hydrophobicity or fungal development, but others include attaching to host surfaces [62] or activating signaling pathways in plants [63]. The type II hydrophobin coding gene *tvhydii1* played a role in plant root colonization and mycoparasitism, and its expression was induced not only in the presence of *A. thaliana*, but also in the presence of the phytopathogen *R. solani* [14]. The gene *hfb7* from *T. virens*, which codes for a type II hydrophobin, is strongly induced in the presence of the plant pathogens *F. oxysporum* f.sp. *cubense* 4 (FOC4) and *Sclerotium rolfsii*, and it is also induced in the presence of tomato roots [64], suggesting that type II hydrophobins play a role in the interactions of *T. virens* with fungal and plant hosts, but there is no evidence of their expression or role in interactions with PGPBs. However, our results showed that *tvhydii1* was downregulated in the presence of the pathogen *F. brachygibbosum* and most of the PGPBs; only when *T. virens* was growing along with Fb and AF12 did this gene increase its expression (Figure 6B), on the 3rd day of confrontation, indicating the possibility that this gene is involved at early stages of the interaction with *B. velezensis* AF12 and *F. brachygibbosum*. Meanwhile, the expression of *tvhydii1* was not significantly different in the presence of the plant and PGPBs than when Tv was growing alone in our plant growth-promotion assays (Figure 7C), suggesting that it may not be involved in the tripartite interaction *T. virens*–plant–PGPB.

Considering our results from the expression, we observed that the effector coding genes that we selected are not involved in the communication between *T. virens* and the PGPBs we used and may not be responsible for the resulting interactions with both the plat pathogen and the plant, but rather the secondary metabolites from each other microorganisms may be the ones driving the interactions, since secreted compound had a strong inhibitory effect (Figure 1).

Taken together, the synergistic effects between *T. virens* and the evaluated PGPBs were strain- and host-specific, and the selected effector-coding genes are not involved in these interactions. However, we do not rule out that other effector genes may still contribute to their establishment. Tv + SER3 and Tv + AF12 showed greater inhibition of *F. brachygibbosum* than individual strains, whereas Tv + AF23 did not differ from single inoculations in restricting pathogen growth. Similarly, although Tv + PGPB consortia improved plant biomass and root development, they were not superior to the best-performing single strains, particularly UM270, SER3, and AF12. Overall, the lack of synergy in Tv + AF23 highlights the need to carefully dissect interactions among beneficial microorganisms when designing microbial consortia for biocontrol or plant biostimulation. This study underscores that interactions between plant-beneficial microorganisms can yield outcomes that differ from expected or previously reported effects.

## 4. Materials and Methods

### 4.1. Organisms, Growth and Culture Conditions

*T. virens* Gv29-8 (referred to as Tv in this study) was provided by Dr. Alfredo Herrera-Estrella’s laboratory at Unidad Genómica Avanzada (Cinvestav-Irapuato). *Arabidopsis thaliana* Col-0 seeds were provided by Dr. Paulina Guzmán-Guzmán. *Pseudomonas fluorescens* UM270 (referred to as UM270 in this study), *Rouxiella badensis* SER3 (referred to as SER3 in this study) and *cillus velezensis* AF12 (referred to as AF12 in this study), *Bacillus halotolerans* AF23 (referred to as AF23 in this study), and *Fusarium brachygibbosum* (referred to as Fb in this study) were strains isolated by Dr. Santoyo’s research group from different sources and are part of Dr. Santoyo’s laboratory strain repository [27,65,66].

To obtain conidia from *T. virens* Gv29-8 (Tv), the strain was inoculated in Petri dishes containing potato dextrose agar medium (PDA) and incubated at 28 °C for 7 to 10 days until full conidiation [14]. Conidia were collected with sterile distilled water and filtered using Magitel^®^ filters and counted using a Neubauer chamber. Then, the total conidia count was adjusted to a concentration of 1 × 10^6^ conidia mL^−1^ for the experiments. Four plant growth-promoting bacteria were used in this study: *P. fluorescens* UM270 (UM270) [16], *R. badensis* SER3 (SER3) [27], *B. velezensis* AF12 (AF12), and *B. halotolerans* AF23 (AF23) [66]. Each PGPB strain was grown in nutrient broth (NB) for 24 h at 30 °C. Serial dilutions of each liquid culture were prepared and plated on Petri dishes containing nutrient agar medium (NA) and incubated overnight at 30 °C. Bacterial colonies were counted to determine the colony-forming units (CFU) of each PGPB and then were adjusted to a concentration of 1 × 10^6^ CFU mL^−1^ to use in the experiments. Actively growing mycelia from the plant pathogen *F. brachygibbosum* strain 4BF (access number MN365015.1) [27] were obtained by inoculating the strain in Petri dishes with PDA and incubated at 28 °C in darkness until full coverage of the plate was achieved. Fresh cultures of each microorganism were prepared before the experiments. *A. thaliana* Col-0 (At) seeds were sterilized with 96% ethanol five times, then air-dried in a laminar flow chamber and kept in sterile conditions until use in experiments. Before any experiment was conducted, *Arabidopsis* seeds were vernalized for 48 h at 4 °C in the dark [17].

### 4.2. Consortia Development of T. virens and PGPBs and Co-Inoculation Procedures

Four combinations of *T. virens* with each of the PGPBs were established as follows: *T. virens* + *P. fluorescens* UM270 (Tv + UM270), *T. virens* + *R. badensis* SER3 (Tv + SER3), *T. virens* + *B. velezensis* AF12 (Tv + AF12), and *T. virens* + *B. halotolerans* AF23 (Tv + AF23). Microorganisms were inoculated in the same Petri dish according to the combinations at the time of the experiments. Fresh cultures of each microorganism were prepared 24 h before the experiment. In the co-inoculation experiments, an aliquot of 1 × 10^6^ CFU of PGPB was placed along the bottom of the plate, and at one side of the PGPB inoculum, either a plug containing actively growing mycelia of *T. virens* or an aliquot containing 1 × 10^6^ conidia was placed [17], thus establishing the combinations of microorganisms.

### 4.3. Compatibility Essays of T. virens and PGPBs

Four combinations of microorganisms were co-inoculated in Petri dishes containing PDA or MS medium to test if *T. virens* could grow and/or germinate in the presence of each of the PGPB used in this work, Each PGPB strain was inoculated in a cross shape along the medium, dividing the plate into four quadrants, and in each of the quadrants an inoculum of 1 × 10^6^ conidia of *T. virens* was placed, or a plug of PDA containing actively growing mycelia (Supplemental Figure S1). Plates were incubated at 30 °C and observed for 3 days to determine if bacteria and fungus could grow together, and conidial germination was not inhibited by the bacteria [17]. The experiment was repeated thrice, with similar results.

Additionally, it was tested whether the secondary metabolites secreted by each of the microorganisms could inhibit each other and help with the selection of the best consortium [17,24]. The experiment was conducted as follows: a sterile cellophane sheet was placed over PDA medium, and either *T. virens*, *F. brachygibbosum,* or a PGPB was inoculated on the cellophane and incubated for 3 days. After the incubation period, the cellophane was removed, along with the growing microorganism. Each of the PGPB, *T. virens* or *F. brachygibbosum* was then inoculated on the medium without cellophane and incubated for 7 days to observe their growth over the medium containing secondary metabolites from the previous microorganism. The control conditions were the microorganisms growing alone without secreted compounds. This experiment was repeated twice with three individual replicates, and similar results were obtained.

### 4.4. Biocontrol Assays with T. virens and PGPBs

Biocontrol experiments were performed in Petri dishes containing Potato Dextrose Agar medium (PDA). On one side of the plate, a plug containing actively growing mycelium from the pathogen *F. brachygibbosum* was placed at 2 cm from the edge. At the opposing side of the plate, an inoculum containing 1 × 10^6^ CFU of each PGPB, 1 × 10^6^ conidia from *T. virens* or both inoculums in the case of the consortia were placed at 2 cm from the edge Then, plates were incubated in total darkness at 30 °C for 7 days to determine the percentage of pathogen growth inhibition at the 3rd, 5th, and 7th day of confrontation. Photographs were taken at each time point, and the pathogen colony area was measured with ImageJ^®^ software (version 1.54d). The control condition was the pathogen growing alone. Three independent biocontrol experiments were performed, and similar results were obtained.

The percentage of pathogen growth inhibition was calculated with the following formula [17]:

$$\%\;GI=\frac{{Fb}_{control}-{Fb}_{treatment}}{{Fb}_{treatment}}\times 100$$

where %GI is the percentage of growth inhibition of *F. brachygibbosum*, Fb_control_ is the colony area of the pathogen growing alone and Fb_treatment_ is the colony area of the pathogen growing in confrontation with the single beneficial microorganisms or in consortium.

### 4.5. Plant Growth Promotion Experiments with T. virens and PGPBs

Plant growth promotion experiments were performed in Petri dishes containing 0.2× Murashige–Skoog medium (MS) supplemented with 1 g/L of MES as pH buffer [67]. Each plate was inoculated with 10 sterilized *A. thaliana* Col-0 seeds (see Section 4.1) and vernalized at 4 °C for 48 h. Then, the plates containing the seeds were incubated in a plant growth chamber at 22 °C and 16 h light/8 h darkness conditions for germination. On the 4th day of germination, the microorganism consortium or the microorganisms alone were placed at 5 cm from the plant roots, applying a concentration of 1 × 10^6^ CFU of PGPB and/or placing an inoculum of 1 × 10^6^ conidia from *T. virens*. The interactions were performed for five days, and primary root length, plant height, fresh and dry weight, and number of lateral roots were measured on the 3rd and 5th day of interaction. Plants growing alone were used as a control condition. Three independent experiments were conducted, and similar results were obtained.

### 4.6. Relative Expression of T. virens Genes sm1, tvsep3, tvhydii1

Biocontrol assays and plant growth-promotion experiments were performed as indicated above, but a sterile cellophane sheet was placed over the medium prior to inoculation of *T. virens* to collect mycelia from the front of the actively growing *Trichoderma* colony (0.5 cm). The collected mycelia were placed inside a microcentrifuge tube and frozen in liquid nitrogen immediately. Samples were kept at −80 °C until processing. Total RNA was extracted using the TriZol^®^ protocol, and RNA integrity was verified by agarose gel electrophoresis. Total RNA was quantified with a Nanodrop (Thermo Scientific, Waltham, MA, USA), and then cDNA synthesis was performed using GoScript™ Reverse Transcriptase (Promega), according to the manufacturer’s protocol. A qRT-PCR cycling program was performed as follows: 95 °C hold for 5 min, followed by 40 cycles of denaturation at 95 °C and annealing and extension at 62 °C; a melt curve was included to ensure single specific amplicons for all primers used. qRT-PCR was performed using SybrGreen master mix (Radiant™) with 20 ng of cDNA as template for each reaction. The housekeeping gene used as an expression control was the glyceraldehyde-3-phosphate dehydrogenase gene (*gpd*) from *T. virens*, and three replicates from each treatment were analyzed by the ΔΔCt method to determine relative expression of the genes *sm1*, *tvsep3*, and *tvhydii1* using StepOne software v2.3 (Applied Biosystems). The control conditions were the fungus growing alone. Primers used in this study are described in Table S1 and were previously designed and tested [14]. All raw data are available on reasonable request to the corresponding author.

### 4.7. Statistical Analysis

A two-way ANOVA with Dunnett’s *post hoc* test of mean comparison was used to analyze the qRT-PCR and plant growth promotion results. A one-way ANOVA with Tukey’s post hoc test was used to analyze the results of the biocontrol assays. All statistical analyses were performed using GraphPad Prism 8 software (GraphPad Inc.).

Compatibility assays of *T. virens* and PGPBs were repeated three times independently, and five samples of each treatment were used in each biological replicate (*n* = 15). Three independent biocontrol experiments were performed using three individual replicates per treatment/per independent experiment (*n* = 9). Three independent plant growth promotion experiments were carried out, using three individual replicates per treatment/per independent experiment, each containing 10 *A. thaliana* seedlings (*n* = 90).

## 5. Conclusions

Although consortia of plant growth-promoting bacteria (PGPB) have been proposed as promising strategies for biocontrol and biostimulation, little is known about their interactions with the beneficial fungus *Trichoderma*. In this study, several combinations of *T. virens* and PGPB showed enhanced biocontrol activity against *F. brachygibbosum* and promoted plant growth, whereas others exhibited effects like the uninoculated control or were less effective than individual inoculations. These results highlight the need to better understand consortium assembly, including aspects such as inoculation timing, for example, whether PGPB should be inoculated before *Trichoderma* to allow their establishment in the plants and to avoid inhibition by metabolites secreted by both species, so that microbe-microbe interactions improve their establishment and their impact on plant performance. Overall, this work underscores the importance of understanding the interactions among beneficial microorganisms, as the results observed *in vitro* suggest that these interactions could influence their capacity for biocontrol and plant growth promotion under natural conditions.

## Data Availability

All raw data are available on reasonable request to the corresponding author.

## Supplementary Materials

The following supporting information can be downloaded at https://www.mdpi.com/article/10.3390/plants15142234/s1, Figure S1: Growth compatibility of *T. virens* and PGPBs. Growth compatibility of *T. virens* and PGPBs. Each PGPB was streaked in a Petri dish containing PDA medium; in each quadrant of the plate, plugs of actively growing mycelia (upper left and bottom right quadrants, bottom line) and 1 × 10^6^ conidia from *T. virens* (upper right and bottom left quadrants, bottom line) were inoculated. The experiment was performed in triplicate. The control condition was the growth of microorganisms alone. The experiment was performed three times, with similar results; Table S1: Primers used in this study and size of the amplicons.

## Abbreviations

The following abbreviations are used in this manuscript:

Tv	*Trichoderma virens*
UM270	*Pseudomonas fluorescens* UM270
SER3	*Rouxiella badensis* SER3
AF12	*Bacillus velezensis* AF12
AF23	*Bacillus halotolerans* AF23
Fb	*Fusarium brachygibbosum*
At	*Arabidopsis thaliana*
