# Supplementary material for: Synergistic Interactions Between Plant Growth-Promoting Bacteria and Trichoderma virens Enhance Arabidopsis Growth and Suppress Fusarium brachygibbosum
Source: Plants (Basel). 2026 Jul 22;15(14):2234. doi: 10.3390/plants15142234 (PMC13415227; doi:10.3390/plants15142234)
Supplement: Supplementary file 1 [file plants-15-02234-s001.zip › plants-4427605-supplementary.pdf]

Table S1. Primers used in this study and size of the amplicons.

List of primers used for this study			
**Name**	**Sequence 5'→3'**	**Use**	**Fragment**
Tvhydii1 AS	TCAGTGCAGAGAACAGCAACTCCA	qRT-PCR for gene *tvhydii1*	95bp
Tvhydii1S2TR	AAATGCAAGTCGTTTGGCAGCGTC		
Sm1cs1	CTGCCGCCGTCTCCGCGGACAC	qRT-PCR for gene *sm1*	223bp
Sm1as1	CTGTATTGCAGCTTCCAGCA		
Tvsep3AS	ATACCACGCAGTCCCATCAATCCA	qRT-PCR for gene *tvsep3*	124bp
Tvsep3S2TR	TCTGTTGTCGAAGCCCAACAGAGA		
GPD-F	GCTGCCGATGGTGAGCTCAAGGG	qRT-PCR for the housekeeping gene *gpd*	132bp
GPD-R	GAGGTCGAGGACACGGCGGGA		

Suppl. Figure S1.

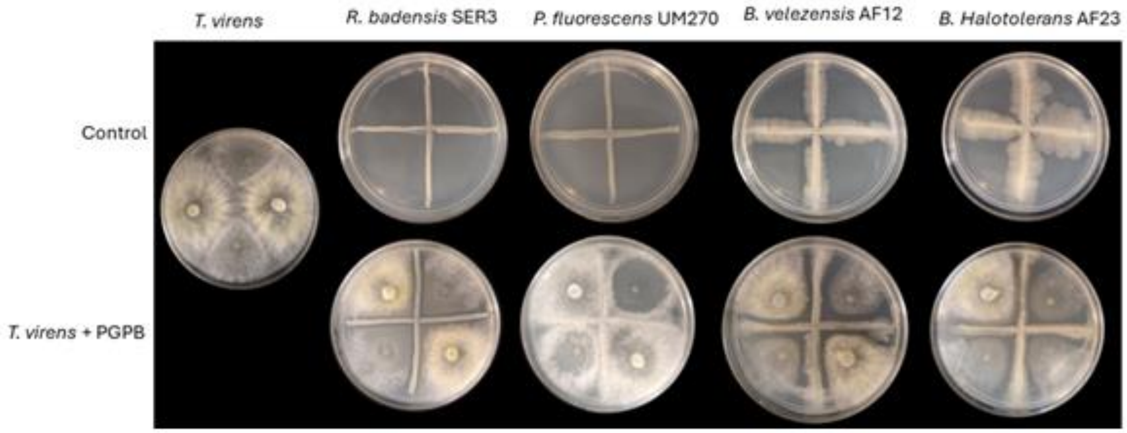

$$\text{Synergistic/Antagonistic Effect (\%)} = \frac{\text{Consortium Activity} - \text{Summed Isolate Activity}}{\text{Summed Isolate Activity}} \times 100$$

Plant Growth Promotion Experiment										
Synergistic/Antagonistic effect (100%)										
Consortium	Primary Root length		Plant Height		Number of Lateral Roots		Fresh Weight		Dry Weight	
	3dpi	5dpi	3dpi	5dpi	3dpi	5dpi	3dpi	5dpi	3dpi	5dpi
Tv + UM270	-72.09	-62.59	-100.00	-47.83	-16.67	-32.69	-73.90	-84.26	-60.00	-84.44
Tv + SER3	-44.81	-50.05	-51.28	18.52	50.00	50.00	-54.33	-67.54	0.00	-60.87
Tv + AF12	-45.40	-51.71	-40.00	15.63	0.00	58.82	-40.09	-77.89	16.67	-89.74
Tv + AF23	-28.14	-53.40	-33.33	33.33	-300.00	-7.14	-47.89	-49.48	-35.71	-65.63

F. brachygibbosum Growth Inhibition Experiment			
Synergistic/Antagonistic effect (100%)			
Consortium	3dpi	5dpi	7dpi
Tv + UM270	-9.14	12.28	5.81
Tv + SER3	10.13	-27.75	-19.42
Tv + AF12	33.64	-43.30	-8.56
Tv + AF23	-9.75	-7.39	-57.61
